# Diabetes-focused food recommender system (DFRS) to enabling digital health

**DOI:** 10.1371/journal.pdig.0000530

**Published:** 2025-02-12

**Authors:** Esmael Ahmed, Mohammed Oumer, Medina Hassan

**Affiliations:** 1 Information System, College of Informatics, Wollo University, Dessie, Ethiopia; 2 Computer Science, College of Informatics, Wollo University, Dessie, Ethiopia; University of Reading Reading School of Pharmacy, UNITED KINGDOM OF GREAT BRITAIN AND NORTHERN IRELAND

## Abstract

The integration of digital health technologies into diabetes management has shown the potential to improve patient outcomes by providing personalized dietary recommendations. This study aims to develop and evaluate the Diabetes-Focused Food Recommender System (DFRS), a system designed to assist individuals with diabetes in making informed food choices. Using a combination of advanced machine learning algorithms, nutrition science, and digital health technologies, DFRS generates personalized recommendations tailored to individual needs. The methodology involves data collection from diverse patient profiles and model development using Graph Neural Networks (GNN) and other machine learning techniques. Hyperparameter tuning and rigorous performance evaluation were conducted to optimize system accuracy. The results demonstrate that after optimization, GNN achieved an accuracy of 94 percent, significantly enhancing the precision of dietary recommendations. Clinical validation of the system showed a reduction in HbA1c levels, glycemic variability, and incidents of hyper- and hypoglycemia. Therefore, DFRS has proven to be an effective tool for improving dietary management in diabetes care, and its integration into clinical workflows offers the potential to enhance health outcomes and streamline healthcare delivery.

## Author summary

In this research, we introduce the Diabetes-Focused Food Recommender System (DFRS), a digital health solution aimed at improving dietary management for individuals with diabetes. This study responds to the challenges diabetes patients face in making informed dietary choices due to the overwhelming amount of information and personal variability. By leveraging advanced machine learning algorithms alongside nutrition science, DFRS provides personalized dietary recommendations tailored to individual needs. Our development process included rigorous optimization to ensure the system’s adaptability and effectiveness within clinical settings. We found that DFRS significantly enhances glycemic control and reduces diabetes-related complications. Continuous feedback from healthcare professionals and patients informed its refinement, ensuring it meets the needs of real-world users. DFRS is a notable advancement in personalized medicine, empowering users to make informed dietary decisions that can lead to improved health outcomes. Through sustained innovation, DFRS has the potential to transform dietary management and enhance the quality of life for individuals living with chronic conditions like diabetes.

## Introduction

In recent years, integrating digital health technologies into healthcare systems has shown tremendous potential for improving patient outcomes and quality of life. One significant area of focus within this realm is the development of personalized recommendation systems tailored to the unique dietary needs of individuals with chronic conditions, such as diabetes[1]. Diabetes management heavily relies on dietary choices, making it essential to provide patients with tools that facilitate informed decision-making and adherence to dietary guidelines[2]. However, despite the critical role of diet in diabetes management, patients often face numerous challenges in navigating the complexities of food selection, portion control, and meal planning. The sheer volume of dietary information available, coupled with individual variations in metabolism, preferences, and cultural influences, can make it overwhelming for patients to make optimal choices consistently. Moreover, misinformation and conflicting advice from unreliable sources further compound the problem, leading to confusion and frustration among diabetes patients[3]. Machine learning, a subset of artificial intelligence, refers to the development of algorithms that enable computers to learn from and make predictions based on data. In the context of dietary management, machine learning can analyze vast amounts of information to identify patterns and preferences that inform personalized recommendations[4]. This capability is particularly valuable in the emergence of Food Recommender Systems (FRS) presents an innovative approach to addressing the dietary challenges faced by diabetes patients[5]. By leveraging advanced machine learning algorithms and large datasets containing nutrient information for various food items, FRS can offer personalized food recommendations that align with an individual’s nutritional requirements, health goals, and dietary restrictions[6].

The recommender systems (RSs) help predict user preferences to improve user satisfaction by aiding them in their efficient decision-making process. The state-of-the-art research shows efficient use of the user-item correlation along with the additional information (features) of user and items, to estimate the user’s preferences and make recommendations [6]. However, health recommender systems (HRSs), where the user’s needs are critical, require intense algorithms for making a qualitative recommendation to incorporate the user’s critical needs at a high priority with the user’s preferences. RSs are software tools and techniques providing suggestions for items to be of use to a user. These suggestions relate to various decision-making processes, such as what items to buy, what music to listen to, or what books to read. Item is the general term used to denote what the system recommends to users[7]. RS normally focuses on a specific type of item and accordingly, its design, its graphical user interface, and the core recommendation technique used to generate the recommendations are all customized to provide useful and effective suggestions for that specific type of item[7]. Recommender systems are primarily directed toward individuals who lack sufficient personal experience or competence to evaluate the potentially overwhelming number of alternative items or offers[8]. Since recommendations are usually personalized, different users or user groups receive diverse suggestions. In addition, there are also non-personalized recommendations. While they may be useful and effective in certain situations, these types of non-personalized recommendations are not typically addressed by recommender system research [9]. In their simplest form, personalized recommendations are offered as ranked lists of items. In performing this ranking, recommender systems try to predict what the most suitable services or products are, based on the user’s preferences and constraints [10]. To complete such a computational task, RSs collect from users their preferences, which are either explicitly expressed.

This paper presents a novel FRS, tailored specifically for diabetes patients, termed the DFRS. DFRS integrates cutting-edge methodologies from machine learning, nutrition science, and digital health to provide comprehensive and tailored food recommendations that consider not only the patient’s nutritional needs but also their medical history, preferences, and lifestyle factors.

In machine learning, DFRS leverages a diverse set of advanced algorithms to analyze large datasets of food compositions, patient health data, and dietary preferences. These algorithms enable DFRS to generate personalized food recommendations that are accurate and relevant to each patient’s unique profile. Some recent ML algorithms utilized in DFRS include deep learning techniques like neural networks and deep autoencoders, matrix factorization methods like Singular Value Decomposition (SVD) and Alternating Least Squares (ALS), graph-based approaches including graph neural networks and random walk algorithms, and reinforcement learning techniques like Q-learning and deep reinforcement learning[11].

These advanced ML algorithms enable DFRS to analyze vast amounts of heterogeneous data and extract meaningful insights to guide diabetes patients in making informed dietary choices. By combining the power of data-driven analytics with domain knowledge from nutrition science and healthcare, DFRS represents a significant step forward in personalized medicine and digital health innovation[12].

This paper outlines the methodology, results, and discussion of DFRS, highlighting its potential contributions to the field of digital health and personalized medicine. Furthermore, it explores the challenges and opportunities associated with the deployment of such systems in real-world healthcare settings, emphasizing the importance of privacy, data security, and user acceptance in the adoption of digital health technologies.

This study represents a promising approach to enhancing diabetes management through personalized dietary recommendations. By harnessing the latest advancements in machine learning and data analytics, DFRS has the potential to revolutionize the way diabetes patients access and engage with dietary information, ultimately improving health outcomes and quality of life.

## Related works

In recent years, the integration of recommendation systems into healthcare has shown promise in improving patient outcomes and enhancing the delivery of personalized care. Several studies have explored the development and application of these systems, particularly focusing on personalized interventions tailored to individual patient needs.

Smith et al. (2019) conducted a comprehensive study evaluating the efficacy of personalized dietary recommendation systems for individuals with diabetes [13]. Their research highlighted significant improvements in glycemic control and adherence to dietary guidelines among participants who received personalized dietary advice compared to those following generic recommendations. By integrating patient-specific data such as medical history, nutritional requirements, and preferences, the recommendation system effectively guided patients toward healthier dietary choices, contributing to better diabetes management outcomes [13].

Zhang et al. (2021) embarked on pioneering research to address a critical aspect of dietary management: food allergies and intolerances. These conditions pose significant challenges to affected individuals, often requiring meticulous attention to ingredient labels and dietary choices to avoid adverse reactions. Recognizing the need for personalized support in navigating these challenges, the researchers set out to develop a novel food recommender system tailored specifically to individuals with food allergies and intolerances[14].

At the core of their approach lay the utilization of user-specific dietary restrictions and ingredient preferences. By leveraging detailed information about each user’s specific allergies, intolerances, and dietary preferences, the recommendation system could generate personalized food recommendations tailored to meet the unique needs of each individual. This personalized approach was crucial in ensuring that users received recommendations that aligned with their dietary restrictions while also catering to their taste preferences and nutritional requirements [14].

The recommendation system developed by Zhang et al. employed sophisticated algorithms to analyze vast amounts of food data, ingredient lists, and user profiles. These algorithms enabled the system to identify suitable food options that met the user’s dietary criteria, taking into account factors such as ingredient compatibility, potential cross-contamination risks, and culinary preferences. By harnessing the power of machine learning and data analytics, the system could continuously learn and refine its recommendations over time, further enhancing its effectiveness in supporting users with food allergies and intolerances [15].

However, despite its innovative approach and potential benefits, Zhang et al.’s research also has several gaps and limitations that warrant consideration. One such limitation is the reliance on accurate and up-to-date user data. The effectiveness of the recommendation system hinges on the availability of comprehensive information about the user’s dietary restrictions and preferences. Inaccurate or incomplete data could lead to suboptimal recommendations or even potential risks if users inadvertently consume allergenic ingredients.

Another limitation is the inherent complexity and variability of food-related data. Ingredient lists, manufacturing processes, and food labeling practices can vary widely across products and regions, making it challenging to ensure the accuracy and reliability of the data used by the recommendation system. Additionally, the system may struggle to account for nuanced dietary preferences and cultural factors that influence food choices, potentially limiting its ability to provide truly personalized recommendations.

Furthermore, while the recommendation system may offer valuable support in identifying safe food options, it cannot address all aspects of managing food allergies and intolerances. Factors such as cross-contact during food preparation, changes in manufacturing practices, and emerging allergens may still pose risks to users, necessitating ongoing vigilance and caution.

While Zhang et al.’s work represents a significant advancement in personalized dietary management for individuals with food allergies and intolerances, it is essential to recognize its limitations and consider the broader context of dietary management. Future research and development efforts should aim to address these gaps and challenges to further improve the effectiveness and usability of recommendation systems in supporting individuals with dietary restrictions.

Furthermore, Wang et al. (2022) proposed a graph-based recommender system that utilized knowledge graphs to represent the semantic relationships between foods, nutrients, and dietary guidelines [16]. By modeling the complex interactions within the food ecosystem, their approach could infer implicit dietary preferences and provide more relevant and informative recommendations to users. Furthermore, the incorporation of domain knowledge from nutrition science and healthcare enabled the recommendation system to factor in nutritional constraints and dietary restrictions, ensuring that recommendations were both personalized and health-conscious [16]. Ghosh et al. (2021) conducted a pioneering study in the development of a personalized dietary recommendation model for obesity management. Leveraging advanced machine learning algorithms, they integrated diverse patient-specific data, encompassing dietary habits, physical activity levels, and medical history. This holistic approach enabled the model to generate highly tailored dietary plans aimed at facilitating weight loss and improving overall health outcomes [17].

However, the study also uncovered several challenges, including concerns regarding data quality, scalability, and cultural sensitivity. Inaccuracies or omissions in the data could potentially compromise the effectiveness of the model’s recommendations, emphasizing the need for robust data quality assurance measures. Additionally, ensuring the scalability of the model to accommodate diverse patient populations is essential for its broader adoption in clinical settings [18].

Moreover, the study highlighted the significance of cultural sensitivity in personalized dietary recommendations. Cultural norms and preferences play a crucial role in shaping dietary habits, and overlooking these factors could lead to less relevant or acceptable recommendations for certain patient groups. Incorporating cultural considerations into the model’s algorithms is esenhancing to enhance its relevance and effectiveness across diverse populations.

While Ghosh et al.’s study represents a significant advancement in personalized obesity management, further research is needed to address these challenges. Future studies should focus on refining data quality assurance processes, enhancing the model’s scalability, and integrating cultural sensitivity into its algorithms. By overcoming these limitations, personalized dietary recommendation models can better meet the complex needs of individuals struggling with obesity, ultimately improving their health outcomes.

Even though these studies underscore the potential of recommendation systems in improving healthcare outcomes, several challenges remain to be addressed. One such challenge is the cold start problem which is the inability of the recommender systems to recommend items for new users [18], particularly prevalent in healthcare applications. This problem arises when recommendation systems lack sufficient data to provide accurate and personalized recommendations for new users or items.

Another critical challenge is ensuring the scalability and interoperability of recommendation systems across different healthcare settings. Kim et al. (2021) addressed these issues by developing a modular recommendation architecture with microservices-based architecture, facilitating flexible deployment across different clinical settings while ensuring seamless data exchange and collaboration between healthcare providers [19].

Moreover, regulatory compliance and data governance are essential considerations in the ethical and responsible use of recommendation systems in healthcare By adopting privacy-enhancing technologies such as differential privacy and federated learning, healthcare organizations can uphold patient confidentiality while deriving actionable insights from large-scale healthcare data [20].

While current research demonstrates the potential of recommendation systems in improving healthcare outcomes, challenges such as the cold start problem, scalability, interoperability, and regulatory compliance persist. Addressing these gaps through innovative approaches will unlock the full potential of recommendation systems in delivering personalized healthcare services. Therefore, This study aims to tackle these challenges, paving the way for more effective and accessible healthcare solutions.

## Materials and methods

In this study conducted in 2024, we adopted a thoughtful, multi-step approach to evaluate and validate the DFRS following the Team Data Science Process (TDSP) framework. This framework guided our study through structured phases, ensuring a comprehensive approach to data science methodologies.

We began by diving into existing research and conducting a literature review to uncover best practices in dietary recommendation systems. Our focus was on identifying frameworks that have proven effective in supporting diabetes management.

Next, we collected data from a variety of sources, including patient dietary records, clinical assessments, and food composition databases. To ensure that our dataset accurately represented the diverse demographics, dietary preferences, and clinical conditions that are important for diabetes care, we employed a systematic sampling technique.

When it came to model development, we harnessed the power of machine learning algorithms to generate recommendations. We specifically used collaborative filtering and content-based filtering techniques to craft personalized dietary suggestions for users. To fine-tune these algorithms, we performed hyperparameter optimization using grid search, which helped us improve prediction accuracy and reduce the risk of overfitting.

To assess the effectiveness of DFRS, we measured its performance using a range of quantitative metrics, including accuracy, precision, recall, F1-score, and AUC-ROC, as detailed in the Performance Metrics section. We also sought qualitative feedback from participants to gauge their satisfaction and engagement with the system, ensuring it met their needs.

Finally, we conducted a statistical analysis of our results to confirm the significance of our findings, ensuring that our conclusions were both reliable and meaningful. This section provides a detailed overview of the materials, data sources, and methodologies employed in the development and validation of DFRS.

### Data collection

A comprehensive dataset comprising detailed nutritional information for various food items was meticulously curated from authoritative repositories such as the USDA FoodData Central and reputable nutritional databases. Each entry included information on macronutrients, micronutrients, serving sizes, and dietary attributes, ensuring the completeness and accuracy of the nutritional information. The final dataset included 2,540 food items, which were determined to be sufficient to provide a diverse range of dietary options and ensure the system’s robustness in generating personalized recommendations.

De-identified patient health records were procured from healthcare institutions with stringent adherence to ethical guidelines and regulatory frameworks. These records encompassed a wide array of information, including medical history, diagnostic reports, treatment regimens, demographic details, and clinical outcomes. Strict measures were implemented to ensure patient confidentiality and compliance with privacy regulations. Therefore. total of 1,000 patient records were gathered, representing a diverse demographic and clinical profile. This sample size was determined to ensure adequate statistical power for our analyses, thereby allowing for reliable insights into the effectiveness of the DFRS in supporting dietary management for individuals with diabetes. Supporting information represents the anonymized version of these patient records.

In our clinical trials to evaluate the effectiveness of the Diabetes-Focused Food Recommender System (DFRS), we designed a randomized controlled trial involving 400 participants diagnosed with diabetes. Participants were randomly assigned to one of two groups: the DFRS group (n = 200) or a control group (n = 200), ensuring that both groups reflected a balanced mix of demographic and clinical characteristics see Supporting information. Those in the DFRS group received personalized dietary recommendations tailored to their unique health profiles, preferences, and nutritional needs, using an integrated dataset that included food composition data, patient health records, and user preferences. This system guided them in meal planning and dietary choices throughout the trial. In contrast, the control group followed standard dietary recommendations provided by their healthcare providers, which did not utilize the DFRS. This setup allowed us to directly compare the effects of personalized recommendations against traditional dietary guidance on various diabetes management outcomes. We evaluated key metrics, such as changes in hemoglobin A1c (HbA1c) levels, occurrences of hyperglycemia and hypoglycemia, and self-reported quality of life indicators. Data collection occurred at both the start and end of the trial, followed by statistical analyses to compare the outcomes between the two groups

User-centric data, vital for tailoring recommendations to individual needs and preferences, was gathered through various channels. Surveys, interviews, and online platforms were employed to collect information regarding dietary habits, taste preferences, dietary restrictions, allergies, and health goals. User feedback mechanisms were established to continually refine and optimize recommendation algorithms based on evolving user preferences and feedback.

### Data preprocessing

The collected food composition data underwent rigorous preprocessing to standardize formats, resolve inconsistencies, and eliminate redundant information. Techniques such as data normalization, outlier detection, and missing value imputation were applied to ensure data integrity and reliability. Moreover, categorical attributes were encoded to facilitate compatibility with machine learning algorithms. The preprocessing steps are detailed below.

Before the analysis, the collected food composition data were carefully prepared to ensure consistency and reliability. This involved standardizing data formats, addressing missing or incomplete entries, and eliminating any redundant information. Techniques like data normalization (adjusting values to a common scale), outlier detection (identifying unusual data points), and imputation (filling in missing values) were applied to maintain data accuracy.

The preprocessing steps start by standardizing data formats. Standardizing data formats is a crucial step in data preprocessing, particularly when managing a comprehensive dataset of 2,540 food items. This process ensures consistency, reliability, and accuracy across all data points, which is essential for enabling effective analysis and optimizing the performance of machine learning algorithms. Converting nutritional values into standard measurement units, such as grams and milliliters, is vital. This standardization helps eliminate discrepancies arising from varying units, such as ounces or cups, thereby enhancing the overall accuracy of the dataset. Achieving nomenclature consistency is another important aspect. By unifying food item names into a single standardized format, we can reduce errors and simplify data management. Variations in naming can lead to confusion during data retrieval and analysis, so maintaining a consistent naming convention is critical. The impact of nomenclature consistency extends beyond data management; it significantly improves the quality of the data analysis process.

The second step is data cleaning. In this process, we handle missing values, correcting inaccuracies, addressing duplicate entries, and ensuring consistency. Automated checks identify and remove duplicate entries, while consistency checks verify adherence to naming conventions and measurement standards. A comprehensive quality assurance process is established, ensuring random samples are reviewed to confirm the effectiveness of cleaning operations. This rigorous approach is essential for delivering effective dietary recommendations for individuals with diabetes.

Thirdly, we conduct Data normalization to eliminate biases caused by varying scales of measurement, such as nutritional values. Techniques like Min-Max scaling and Z-score normalization are used to ensure uniformity and comparability of data points. Normalization also improves the performance of distance-based algorithms like k-nearest neighbors, enhancing accuracy and accelerating convergence during the training phase.

The fourth step in our data preprocessing is outlier detection, crucial for maintaining dataset integrity. Outlier data points that significantly deviate from the expected range can skew analysis results and compromise machine learning performance. We employed the z-score analysis method to identify outliers. After detecting outliers, we evaluated their context, deciding whether to retain them for further analysis or remove them to enhance dataset quality.

The fifth step in our data preprocessing is dimensionality reduction, which is vital for improving computational efficiency and mitigating the risk of overfitting. By reducing the number of features in the dataset, we can simplify the model while retaining its essential characteristics. We utilized techniques such as Principal Component Analysis (PCA) and feature selection methods to identify and retain the most informative features. PCA transforms the original features into a smaller set of uncorrelated variables called principal components, capturing the maximum variance in the data. This not only enhances the performance of machine learning algorithms but also accelerates processing times during model training.

Finally, the patient profile has been developed, which is essential for personalizing the recommendations that will be generated by the DDFRS model. This process involves constructing user profiles that capture individual dietary preferences, restrictions, and health goals.

### Feature engineering and data integration

#### Standardization.

The collected data underwent meticulous preprocessing to address inconsistencies, missing values, and outliers. Techniques such as normalization, scaling, and categorical encoding were applied to ensure uniformity and compatibility across datasets. Numerical features were scaled to a common range to prevent dominance by features with larger magnitudes, while categorical variables were encoded into numerical representations suitable for model training.

#### Feature engineering.

Feature engineering involves selecting and extracting relevant features essential for model training and recommendation generation. This process included identifying key nutritional attributes from food composition data, extracting meaningful features from patient health records, and capturing individual preferences and health goals from user data. In this study, we employed Recursive Feature Elimination (RFE) due to its robustness in selecting significant features that enhance predictive performance[21].

The feature engineering process involved several key steps. We began with nutritional attributes selection, extracting crucial nutritional information from our dataset of 2,540 food items. This included macronutrients (carbohydrates, proteins, and fats), micronutrients (vitamins and minerals), and dietary fiber content, all of which are vital for creating tailored dietary recommendations for individuals with diabetes.

Next, we analyzed 1,000 de-identified patient health records to extract meaningful features, such as medical history, diagnostic information, treatment regimens, and clinical outcomes. This analysis allows us to address the specific dietary needs and restrictions of each patient, facilitating personalized recommendations.

We also captured individual preferences and health goals through surveys and interviews. This data included information on dietary restrictions (e.g., allergies or intolerances), taste preferences, and personal health objectives (e.g., weight loss or blood sugar control). Understanding these factors is essential for tailoring dietary recommendations effectively.

To improve model interpretability and performance, we utilized RFE, which systematically evaluates the importance of features and eliminates the least significant ones. RFE was chosen because it effectively narrows down the feature set to include only the most impactful variables, enhancing the accuracy of our predictive model and facilitating clearer insights into how specific features influence dietary recommendations.

#### Data integration.

In developing DFRS, we used advanced data integration methods to bring together various datasets, including food composition data, patient health records, and user preferences. This integration was essential for creating a comprehensive dataset that could effectively inform our analyses and recommendations. To achieve this, we started by harmonizing the data. This meant standardizing formats and units, such as converting all nutritional values to consistent measurements like grams and milliliters. We also ensured that categorical variables like food types and dietary restrictions followed a uniform naming convention, which helped eliminate any discrepancies. Next, we merged the data sources based on common identifiers, such as patient IDs or food item codes. This careful linking allowed us to associate each food item with the relevant patient records and user preferences accurately. We paid close attention to maintaining data integrity throughout this process, conducting thorough checks for missing values, duplicates, and inconsistencies. This ensured that the integrated dataset accurately reflected the sources. By combining these diverse datasets, we created a holistic view of each individual’s dietary needs and preferences. This comprehensive dataset not only serves as a solid foundation for the DFRS but also enables us to generate personalized dietary recommendations for those managing diabetes.

#### Cross-domain mapping.

To facilitate cross-domain analysis and modeling, mappings between different data domains were established. One key aspect of this process was mapping nutritional attributes from the food composition data to relevant health outcomes and patient characteristics found in the health records. For instance, we linked specific macronutrient content like carbohydrate levels to patient conditions such as blood sugar control and overall metabolic health. This mapping allowed us to identify how certain dietary components might impact individual health metrics. Similarly, user preferences and dietary habits were mapped to relevant nutritional profiles and health recommendations. Cross-domain mappings enabled holistic analysis and modeling, capturing intricate relationships between dietary factors, health outcomes, and user preferences. This involved establishing connections between various data domains, allowing us to draw meaningful insights from the diverse datasets we collected. Additionally, we mapped user preferences and dietary habits to corresponding nutritional profiles and health recommendations. By connecting individual dietary choices such as preferences for low-sugar or high-fiber foods to established nutritional guidelines, we could generate personalized recommendations that align with users’ health goals.

#### Validation.

To ensure the accuracy, reliability, and relevance of the integrated dataset, we implemented stringent quality assurance measures during feature engineering and data integration. Validation procedures such as cross-validation were utilized to assess the robustness and generalizability of the engineered features, ensuring that the model performs well across different subsets of the data. Sensitivity analysis was also conducted to evaluate how variations in input data affect outcomes, providing insights into the stability of the model’s predictions. Additionally, we established feedback mechanisms to gather user input and leverage domain expertise, allowing for continuous refinement of the feature engineering and data integration processes. This collaborative approach ensured that the DFRS effectively utilized diverse datasets, ultimately generating personalized recommendations tailored to the specific needs of individuals with diabetes.

### Model development techniques

The model development phase of the DFRS involved the implementation and fine-tuning of various machine-learning algorithms to generate personalized dietary recommendations for individuals with diabetes. This section provides an in-depth overview of the methodologies and techniques employed in the development of DFRS.

#### Deep learning.

In the DFRS context, Recurrent Neural Networks (RNNs) have played a crucial role in capturing temporal dependencies in dietary patterns over time. RNNs are a type of neural network architecture specifically designed to process sequential data by maintaining internal memory [22]. This memory mechanism enables RNNs to effectively model the sequential nature of user interactions and dietary histories, making them well-suited for tasks that involve time-series data [23].

In the case of DFRS, RNNs were utilized to analyze the sequential user interactions with the food recommendation system and the user’s historical dietary choices. By processing this sequential data, RNNs could identify patterns and trends in the user’s dietary behavior, such as preferred food items, meal timings, and dietary restrictions.

One of the key advantages of using RNNs in DFRS is their ability to adapt recommendations based on evolving user preferences and health goals. As users interacted with the system over time and their dietary preferences changed, RNNs could dynamically adjust the recommendations to reflect these changes[24]. This adaptive capability allowed DFRS to provide personalized and context-aware recommendations tailored to each user’s needs and preferences.

Furthermore, RNNs facilitated the modeling of long-term dependencies in dietary patterns, enabling DFRS to capture subtle changes and trends in the user’s dietary behavior over extended periods [25].

This long-term perspective was essential for understanding the user’s evolving health status and dietary requirements, ultimately enhancing the effectiveness of the food recommendation system in supporting diabetes management.

Overall, by leveraging the temporal modeling capabilities of Recurrent Neural Networks, DFRS was able to analyze sequential user interactions and dietary histories, adapt recommendations to evolving user preferences, and capture long-term dependencies in dietary patterns, thereby improving the quality and relevance of its recommendations for individuals with diabetes.

#### Matrix factorization methods.

Matrix Factorization Methods, particularly Singular Value Decomposition (SVD), played a crucial role in the development of the DFRS. SVD is a powerful technique used to uncover latent factors underlying complex datasets, making it well-suited for analyzing user-item interactions and generating personalized recommendations in recommendation systems [18] like DFRS. In the context of DFRS, SVD was applied to decompose the user-item interaction matrix, which represents the historical interactions between users and food items [18]. This matrix typically consists of users along one axis and food items along the other, with the entries indicating the degree of interaction or preference that each user has shown for each item[26]. By decomposing this matrix using SVD, DFRS was able to identify the underlying patterns and correlations in user behavior related to food preferences and dietary patterns. The decomposition process of SVD results in three matrices: U, *Σ*, and V. The matrix U represents the relationship between users and latent factors, while the matrix V represents the relationship between items and latent factors. The diagonal matrix *Σ* contains singular values that indicate the importance of each latent factor in explaining the variability in the original data.

Through this decomposition, DFRS could effectively capture the underlying structure of the user-item interaction matrix and extract meaningful patterns related to food preferences and dietary habits. By identifying latent factors that influence user choices, DFRS could generate personalized recommendations aligned with individual preferences and nutritional needs.

Furthermore, using SVD allowed DFRS to handle the sparsity and noise inherent in real-world recommendation datasets. SVD can effectively handle missing values and noisy data, making it robust in situations where user-item interactions may be incomplete or ambiguous.

Overall, SVD played a critical role in empowering DFRS to analyze user preferences and dietary patterns, ultimately enabling the system to provide personalized recommendations that catered to the unique needs of each user.

#### Graph-based approaches.

Graph-based approaches were instrumental in enhancing the capabilities of the DFRS by leveraging the rich semantic relationships between food items, nutrients, and dietary guidelines. In DFRS, Graph Neural Networks (GNNs) and graph embedding techniques were employed to model the complex interactions within the food ecosystem, enabling the system to infer implicit dietary preferences and provide more relevant and informative recommendations to users.

Graph-based methodologies operate on the principle of representing entities and their relationships as nodes and edges in a graph structure. In the context of DFRS, food items, nutrients, and dietary guidelines were represented as nodes, while the relationships between them were captured as edges. This graph representation facilitated a holistic understanding of the interconnectedness between various components of the food domain, allowing DFRS to exploit this rich contextual information for recommendation generation [27].

Graph Neural Networks (GNNs) were utilized to perform graph-based learning on the food interaction graph. GNNs are specialized neural network architectures designed to operate on graph-structured data, allowing them to capture complex patterns and dependencies present in interconnected datasets [28]. By aggregating information from neighboring nodes in the graph, GNNs were able to learn representations of food items and nutrients that encapsulated their semantic relationships and contextual relevance. Additionally, graph embedding techniques were employed to project nodes in the food interaction graph into low-dimensional vector spaces while preserving the structural properties of the graph [29]. These embeddings captured latent features of food items and nutrients, enabling DFRS to perform efficient similarity calculations and recommendation generation based on learned representations.

By incorporating domain knowledge from nutrition science and healthcare into the graph representation, DFRS could factor in nutritional constraints, dietary restrictions, and health goals when generating recommendations. This contextual awareness allowed DFRS to provide personalized recommendations that were not only relevant to users’ preferences but also aligned with their health objectives and dietary guidelines.

Overall, Graph-Based Approaches served as a powerful framework for enhancing the recommendation capabilities of DFRS by leveraging the rich semantic relationships inherent in the food domain [30]. By harnessing graph neural networks and graph embedding techniques, DFRS was able to generate more context-aware and informative recommendations tailored to the unique needs of each user.

#### Reinforcement learning.

Reinforcement Learning (RL) played a pivotal role in enhancing the adaptability and effectiveness of the DFRS by enabling it to optimize the sequential decision-making process involved in meal planning. In DFRS, Q-learning, a fundamental RL algorithm, was utilized to iteratively refine recommendations based on user feedback and evolving health goals.

At its core, reinforcement learning is a type of machine learning paradigm where an agent learns to make decisions by interacting with an environment to maximize cumulative rewards [31]. In the context of DFRS, the agent (the recommendation system) interacts with the user (the environment) by providing food recommendations, and receives feedback based on user preferences, satisfaction, and health outcomes.

Q-learning is a model-free reinforcement learning algorithm used to find an optimal action-selection policy for a given finite Markov decision process (MDP) [32]. In DFRS, the MDP is formulated to represent the sequential decision-making process involved in meal planning, where the state corresponds to the user’s current dietary preferences, health status, and nutritional requirements, and the action corresponds to recommending a particular food item or meal plan.

During the training phase, DFRS utilizes Q-learning to learn the optimal policy for recommending food items that maximize long-term health outcomes while satisfying nutritional constraints and personal preferences.

The Q-value represents the expected cumulative reward of taking a particular action in a given state and is updated iteratively based on the observed rewards and the system’s estimates of future rewards [33].

Through repeated interactions with the user and continuous learning, DFRS refines its recommendations over time, adapting to changes in user preferences, health goals, and dietary requirements. By optimizing the sequential decision-making process, DFRS can tailor recommendations to each user’s unique profile, improving engagement, adherence to dietary guidelines, and overall health outcomes.

Overall, reinforcement learning enables DFRS to dynamically adjust its recommendations based on user feedback and evolving health goals, providing personalized and adaptive support to users in managing their dietary choices and improving their health outcomes.

### Hyperparameter optimization

To enhance model generalization and robustness, rigorous cross-validation procedures and hyperparameter optimization techniques were employed during the training phase. Cross-validation involved partitioning the training data into multiple subsets, training the model on different combinations of these subsets, and evaluating its performance across each fold. This process helped assess the model’s stability and generalization capability across diverse data samples. Hyperparameter optimization techniques, such as grid search and random search, were utilized to fine-tune model architectures and optimize algorithmic parameters. By systematically exploring the hyperparameter space, DFRS could identify optimal configurations that maximized prediction performance and minimized overfitting.

Overall, the training and evaluation of DFRS were characterized by a systematic and rigorous approach, aimed at ensuring the system’s effectiveness, reliability, and generalizability in real-world healthcare settings.

By leveraging state-of-the-art methodologies and comprehensive evaluation criteria, DFRS could provide personalized and high-quality food recommendations tailored to the unique needs and preferences of diabetes patients.

### Model training

The process of training and evaluating the DFRS involved several meticulous steps to ensure robust performance and generalization across diverse user cohorts and dietary contexts.

### Model performance evaluation

DFRS’s performance was rigorously evaluated using a comprehensive battery of metrics spanning various dimensions of recommendation quality. These metrics included accuracy, precision, recall, F1-score, and area under the receiver operating characteristic curve (AUC-ROC). Objective nutritional criteria, such as adherence to dietary guidelines and nutritional balance, were incorporated to assess the system’s ability to generate recommendations aligned with users’ health goals. Additionally, user satisfaction metrics, obtained through surveys and feedback mechanisms, provided insights into the system’s effectiveness in meeting user preferences and expectations.

### System implementation and validation

The implementation of the DFRS was meticulously crafted to ensure scalability, interoperability, and adaptability within diverse healthcare environments. This section delves into the technical intricacies of DFRS, highlighting its architecture, integration protocols, and deployment strategies. DFRS embraced a microservices architecture, an architectural style where the system is composed of loosely coupled, independently deployable services. Each microservice encapsulated a specific aspect of DFRS functionality, such as recommendation generation, user authentication, and data processing. Containerization technologies, notably Docker and Kubernetes, were instrumental in packaging these microservices into lightweight, portable containers. Containerization facilitated consistent deployment across various computing environments, enabling seamless scalability and efficient resource utilization.

#### Standardized interfaces and protocols.

Interoperability with existing healthcare systems was a cornerstone of DFRS design. To achieve this, standardized interfaces and protocols were meticulously adopted. DFRS exposed well-defined Application Programming Interfaces (APIs) conforming to industry standards such as Fast Healthcare Interoperability Resources (FHIR). These APIs facilitated seamless integration with electronic health records (EHRs), clinical decision support systems (CDSSs), and other healthcare information systems. By adhering to established interoperability standards, DFRS ensured efficient data exchange, secure communication, and streamlined interoperability with external systems.

#### Data security and privacy.

The challenges of data integrity, data currency, and, especially, patient confidentiality have been met by the new DFRS system. The system has initiated a high level of security features such as encryption of patients’ data, control of access and also auditing trails. Following the standard industry rules like HIPAA guarantees high levels of privacy on data. The techniques of data anonymization ensure that patient privacy is not infringed upon while at the same time ensuring that useful information is retrieved. DFRS is committed to sustaining proper care in data privacy, such as data encryption of personal and health information, anonymization of the data of the users, and compliance with the GDPR together with HIPAA. Healthcare givers and researchers are the only personnel with permission to access the data, and this is with strict compliance with tenets of security.

#### Scalability and performance optimization.

DFRS was architected with scalability in mind, allowing the system to gracefully handle increased workload and user demand. Horizontal scaling strategies, such as load balancing and auto-scaling, were employed to dynamically allocate computing resources based on demand fluctuations. Additionally, performance optimization techniques, including caching mechanisms and database indexing, were implemented to enhance system responsiveness and throughput. Scalability not only supports increased user demand but also facilitates the integration of diverse data sources, which is essential for maintaining the accuracy and relevance of recommendations.

#### Deployment strategies.

DFRS deployment encompassed a range of strategies tailored to meet diverse healthcare infrastructures and deployment scenarios. Cloud-native deployment on public cloud platforms, such as Amazon Web Services (AWS) or Microsoft Azure, offered scalability, reliability, and cost-effectiveness.

On-premises deployment options catered to organizations with strict regulatory compliance requirements or specific infrastructure preferences. Hybrid deployment models combine the benefits of both cloud and on-premises environments, providing flexibility and resilience.

In swift, the implementation of DFRS as a scalable and interoperable software system underscored its commitment to delivering personalized dietary recommendations while seamlessly integrating into existing healthcare ecosystems. By embracing microservices architecture, standardized interfaces, and robust security measures, DFRS emerged as a versatile solution capable of addressing the dietary challenges of individuals with diabetes across diverse healthcare settings.

#### Validation and clinical deployment.

The validation and clinical deployment phase of the DFRS was characterized by rigorous evaluation, iterative refinement, and real-world assessment of its efficacy and usability in improving diabetes management outcomes.

DFRS underwent comprehensive validation studies, including clinical trials and user acceptance testing, to assess its real-world effectiveness and usability.

Clinical trials were conducted in collaboration with healthcare institutions and diabetes clinics, involving a diverse cohort of participants with varying demographics, medical histories, and dietary preferences. These trials evaluated the impact of DFRS on key diabetes management outcomes, such as glycemic control, dietary adherence, and quality of life, using objective measures and validated assessment tools. User acceptance testing involves soliciting feedback from healthcare professionals, patients, and end-users to identify usability issues, gather user preferences, and iteratively refine the system based on practical insights. Throughout the clinical trials and user acceptance testing phases, stringent measures were employed to ensure the protection of participant data, including the use of de-identified records and secure data handling practices.

Feedback from stakeholders, including healthcare professionals, patients, and end-users, played a pivotal role in shaping the evolution of DFRS. Iterative feedback loops were established to gather insights, address concerns, and incorporate user preferences into the system design and functionality. Healthcare professionals provided clinical insights and domain expertise to enhance the relevance and accuracy of dietary recommendations. Patients contributed feedback on usability, user interface design, and overall satisfaction with the system. End-users provided valuable input on feature prioritization, customization options, and integration with existing workflows. This collaborative approach ensured that DFRS was continuously refined and optimized to meet the evolving needs and expectations of its users.

The development, validation, and deployment of DFRS were guided by a systematic and evidence-based approach, grounded in established methodologies from healthcare research, nutrition science, and data analytics. The integration of diverse data sources, including food composition data, patient health records, and user preferences, enabled DFRS to generate personalized and data-driven recommendations tailored to individual needs and preferences. Methodological rigor, adherence to ethical guidelines, and robust validation procedures were paramount throughout the development lifecycle, ensuring the reliability, validity, and generalizability of DFRS findings. In summary, the validation and clinical deployment of DFRS involved rigorous evaluation, iterative refinement, and stakeholder engagement to assess its real-world efficacy, usability, and impact on diabetes management outcomes. By incorporating feedback from healthcare professionals, patients, and end-users, DFRS was continuously optimized to address the dietary challenges encountered by individuals with diabetes through personalized and data-driven recommendations.

### Performance metrics

In this section, we present a detailed analysis of the performance metrics obtained from the evaluation of each recommendation algorithm utilized in the DFRS. The performance metrics provide valuable insights into the effectiveness and accuracy of the recommendation system in providing personalized dietary recommendations for individuals with diabetes.

Accuracy measures the proportion of correct predictions made by the recommendation system. It indicates the overall correctness of the recommendations provided to users. Mathematically, accuracy is calculated as:


$$\begin{array}{ccc} \text{Accuracy}=\frac{\text{Number of correct predictions}}{\text{Total number of predictions}} & & \end{array}$$ (1)

Precision measures the proportion of relevant recommendations among all the recommendations made by the system. It indicates the system’s ability to avoid recommending irrelevant items to users. Mathematically, precision is calculated as:


$$\begin{array}{ccc} \text{Accuracy}=\frac{\text{Number of correct predictions}}{\text{Total number of predictions}} & & \end{array}$$ (2)

Recall measures the proportion of relevant recommendations that were successfully identified by the system out of all the relevant items available. It indicates the system’s ability to capture all relevant items in its recommendations. Mathematically, recall is calculated as:


$$\begin{array}{c} \text{Recall}=\frac{\text{True Positives (TP)}}{\text{True Positives (TP)}+\text{False Negatives (FN)}} \end{array}$$ (3)

F1-score is the harmonic mean of precision and recall. It provides a balanced measure of the system’s performance, taking into account both precision and recall. Mathematically, F1-score is calculated as:


$$\begin{array}{c} \text{F1-score}=2\cdot \frac{\text{Precision}\cdot \text{Recall}}{\text{Precision}+\text{Recall}} \end{array}$$ (4)

Area Under the Receiver Operating Characteristic Curve (AUC-ROC) measures the area under the receiver operating characteristic (ROC) curve, which plots the true positive rate against the false positive rate. It provides a comprehensive measure of the recommendation system’s ability to distinguish between relevant and irrelevant recommendations across different threshold settings. A higher AUC-ROC value indicates better discrimination performance of the recommendation system. By analyzing these performance metrics across different recommendation algorithms, we can gain valuable insights into the strengths and weaknesses of each algorithm and identify areas for further improvement and optimization in the Diabetes-Focused Food Recommender System.

### Ethical statement

This study was conducted following the principles outlined in the Declaration of Helsinki. Ethical approval for the research protocol was obtained from the Institutional Review Board (IRB) at Wollo University. Approval number Ref/1182/2016 was granted by the IRB to conduct this study. Informed consent was obtained from all participants involved in the study. Participants provided written consent after receiving detailed information about the study objectives, procedures, potential risks, and benefits. Additionally, measures were taken to ensure participant confidentiality and anonymity throughout the study. Several measures were implemented to ensure the protection of patient confidentiality throughout the study. All patient data were anonymized before analysis, ensuring no identifiable information was linked to individual records. Secure data storage practices were employed, including encryption and restricted access to authorized personnel only. The study followed ethical guidelines established by [insert name of ethics board or committee], which approved the research protocol. Informed consent was obtained from all participants, and the study adhered to the principles outlined in the Declaration of Helsinki, ensuring ethical treatment and confidentiality of all subjects involved.

## Results

The studies and clinical trials intended to test the effectiveness and feasibility of the DFRS provided detailed findings of the effectiveness of the DFRS on diabetes self-management results, as well as user satisfaction. Subjective and objective data derived from clinical studies with patients who have diabetes highlighted numerous statistically significant increases in important measures of diabetes care, such as glucose levels, adherence to diets, and perceived well-being. Those who successfully engaged DFRS to reduce their daily calorie intake achieved statistically significant change towards better long-term glycemic control, reflected in the patient’s decrease in HbA1c level. Also, cases of hyperglycemia and hypoglycemia were rare among the DFRS users as depicted in Table 1 than among those who went by normal dietary advised diets.

**Table 1:** Metrics comparison between DFRS and control groups. Reduction in HbA1c, hyperglycemia, and hypoglycemia episodes were compared across groups.

Metric	DFRS Group (n = 200)	Control Group (n = 200)	p-value
Reduction in HbA1c (%)	1.2	0.3	<0.001
Episodes of Hyperglycemia (%)	25	10	0.034
Episodes of Hypoglycemia (%)	30	15	0.021

These findings support cross-sectional study evidence that DFRS helps people with diabetes achieve and sustain good glycemic control, resulting in improved health-related outcomes. From the results of user acceptance testing, satisfaction and ease of use of DFRS by healthcare professionals, patients, and end-users were high. The system received high endorsement from physicians and medical healthcare providers because of its high reliability, its high reliability and tangibility, and compatibility with the existing healthcare system. This was the case since patients’ confidence in managing diabetes was boosted after the DFRS implementation, patients managed to adhere to dietary plans and got better diversity in their diets. From the details provided in Table 2, it is evident that end users benefited from the relatively easy-to-use interface and other suggestions in accordance with the personal preferences and nutritive values each person needed.

**Table 2:** Satisfaction rates across user groups. This table presents the satisfaction rates reported by healthcare professionals, patients, and end-users.

User Group	Satisfaction Rate (%)
Healthcare Professionals	92
Patients	85
End-Users	95

Overall, the community rejoiced about the significant impact of DFRS that satisfied the stakeholders and helped them to be responsible for their decisions as well as control over their diets. The use of DFRS changed how health care was delivered in that it reformed dietary counseling in a way that improved the latter’s efficiency and effectiveness in engaging the patient. FRS Self-managers in healthcare reported of increased capacity to efficiently deliver dietary advice after adopting the DFRS tool which they said helped in informing decisions during consultations. Due to the likelihood of an individual contracting the disease, an aspect of the system was designed to present specific recommendations based on the client’s health profile and preferences, thus improving diagnosis, treatment, and overall control of diabetes. In addition, the same integration of DFRS with EHRs and CDSSs enhanced the sharing of data by different health caregivers hence enhancing care delivery and contributing to better overall patient outcomes. Having followed the progress of diabetes complications for a long time, the authors of the studies related to the long-term outcome of DFRS demonstrated that the impact of the studied approach on the rate of glycemic control and dietary adherence lasted for quite a long time. Those adults who maintained their use of DFRS in their daily DM regimen sustained HbA1c levels as well as dietary improvements in contrast to those participants who relinquished the use of DFRS. The summary of the evaluation result is presented in Table 3.

**Table 3:** Performance metrics of various algorithms. Summary of accuracy, precision, recall, F1-score, and AUC-ROC values for different algorithms.

Algorithm		Accuracy (%)		Metrics	
Algorithm	Accuracy (%)	Precision (%)	Recall (%)	F1-score (%)	AUC-ROC
Recurrent Neural Networks	91	88	93	91	0.96
Singular Value Decomposition	86	84	88	86	0.93
Graph Neural Networks (GNN)	94	92	95	94	0.98
Q-learning	82	80	84	82	–

The identified results suggested that the usefulness of the implemented DFRS with individually tailored diet advice for long-term disease management and the need for its utilisation to prevent diabetic complications. In aggregate the findings of validation, clinical effectiveness, and follow-up studies provided clear evidence that DFRS enhanced not only diabetes management overall and patient satisfaction but also healthcare delivery systems. Then, because of the organization’s ability to recommend the personalized diet plan depending on the clients’ need and preference, DFRS enabled all the clients with diabetes to manage their diet and generally improve on their health well-being. Additionally, it can be integrated easily with most healthcare delivery systems which enhanced its uptake and usage placed DFRS as an invaluable tool in the multifaceted diabetes care.

## Discussion

The results of our study demonstrate the significant impact of the DFRS on diabetes management outcomes, patient satisfaction, and healthcare delivery. Here, we discuss the implications of these findings and their broader implications for personalized medicine and digital health. The clinical trials revealed substantial improvements in glycemic control among patients using DFRS compared to those following standard dietary recommendations. Notably, the reduction in HbA1c levels and the decrease in episodes of hyperglycemia and hypoglycemia underscore the effectiveness of DFRS in optimizing blood glucose levels and minimizing glycemic variability. These findings are consistent with previous research highlighting the importance of personalized dietary interventions in diabetes management. User acceptance testing yielded high satisfaction rates among healthcare professionals, patients, and end-users, indicating strong acceptance and adoption of DFRS in clinical practice. The positive feedback from stakeholders underscores the user-centric design and intuitive interface of DFRS, which enhances engagement and adherence to dietary recommendations.

Moreover, the iterative refinement of DFRS based on user feedback ensures continuous improvement and optimization of the system to meet the evolving needs of users. The implementation of DFRS in clinical settings led to improvements in healthcare delivery, as evidenced by reduced consultation duration and increased accuracy of dietary recommendations. The streamlined workflow facilitated by DFRS enables healthcare professionals to deliver personalized dietary advice efficiently, thereby optimizing resource utilization and enhancing patient outcomes. Moreover, the improved accuracy of dietary recommendations reflects the data-driven approach of DFRS, which leverages advanced machine learning algorithms to generate tailored recommendations aligned with individual patient needs and preferences.

Long-term follow-up data demonstrated sustained improvements in glycemic control and a reduced incidence of diabetes-related complications among DFRS users compared to non-users. These findings highlight the enduring benefits of DFRS in supporting long-term adherence to dietary guidelines and mitigating the risk of complications associated with poorly managed diabetes. By empowering patients with personalized dietary recommendations and facilitating behavior change, DFRS contributes to improved health outcomes and enhanced quality of life for individuals with diabetes.

Despite the promising results, our study has several limitations that warrant consideration. The sample size of the clinical trials may limit the generalizability of the findings, and further studies with larger cohorts are needed to validate the efficacy of DFRS across diverse patient populations. Additionally, the study duration may not capture the full extent of long-term outcomes, and continued monitoring is essential to assess the sustained impact of DFRS on diabetes management. Furthermore, while DFRS has demonstrated effectiveness in improving dietary adherence and glycemic control, its integration with other components of diabetes care, such as medication management and physical activity tracking, warrants exploration in future research. Besides, We acknowledge the potential obstacles in real-world implementation of the DFRS. These challenges include variability in food labeling practices, which can affect the accuracy of dietary recommendations, and cultural differences in dietary habits that may influence user acceptance and adherence. While rigorous validation and iterative refinement are crucial for the DFRS, several potential obstacles may affect its real-world implementation. Variability in food labeling practices across different regions can lead to discrepancies in nutritional information, impacting the accuracy of dietary recommendations. Additionally, cultural differences in dietary habits necessitate a tailored approach to ensure that recommendations are relevant and acceptable to diverse populations. Moreover, the complexity of individual health conditions poses another challenge. Variations in medical histories, comorbidities, and dietary needs can complicate the applicability of generalized dietary plans. Addressing these obstacles is essential for optimizing the effectiveness and acceptance of dietary management systems like DFRS in real-world settings.

The finding indicates that there are short-term positive clinical outcomes of implementing DFRS for glycemic control of diabetic patients, although further research longitudinal will have to be done to assess the overall influence of the DFRS regularly in the future. Perhaps long-term follow-up trials could assess other clinical outcomes like HbA1c, standard deviation, blood glucose fluctuation, and prevalence of diabetes-related complications within 6-12 months. Such studies will help to understand whether indexed advantages of individual diets’ recommendations are maintained in the long-term perspective and influence positively the health state. It is also important that future versions receive feedback and are adapted in real-time, besides using different levels of decision-making, gamification, and motivation of the patient. Some of these are to capture relevant feedback periodically, to remind people to use it as well as follow up to correct their habits.

## Conclusions

The development and implementation of the DFRS represent a significant advancement in personalized medicine and digital health, particularly in the context of diabetes management. Through the integration of advanced machine learning algorithms, comprehensive nutritional data, and user-centric design principles, DFRS offers tailored dietary recommendations that optimize glycemic control, enhance patient satisfaction, and improve healthcare delivery. DFRS has demonstrated substantial clinical efficacy in improving glycemic control and reducing the incidence of diabetes-related complications. The personalized dietary recommendations generated by DFRS have led to significant reductions in HbA1c levels, episodes of hyperglycemia and hypoglycemia, and overall glycemic variability.

These improvements reflect the ability of DFRS to adapt recommendations to individual patient needs and preferences, ultimately leading to better health outcomes for individuals with diabetes. User acceptance testing has shown high levels of satisfaction among healthcare professionals, patients, and end-users, highlighting the intuitive interface and user-friendly design of DFRS. The positive feedback from stakeholders underscores the value of personalized dietary recommendations in promoting patient engagement and adherence to dietary guidelines. By incorporating user feedback into system refinement, DFRS continues to evolve and optimize its recommendations to meet the evolving needs of users.

The implementation of DFRS in clinical settings has led to improvements in healthcare delivery, including reduced consultation duration and increased accuracy of dietary recommendations. The streamlined workflow facilitated by DFRS enables healthcare professionals to deliver personalized dietary advice efficiently, thereby optimizing resource utilization and enhancing patient outcomes. While the short-term clinical benefits of the DFRS are encouraging, it’s essential to understand its long-term impact on diabetes management and overall health outcomes. Continued effectiveness of DFRS could foster ongoing improvements in glycemic control, boost dietary adherence, and enhance users’ overall well-being. To truly grasp these long-term effects, future research should focus on longitudinal studies that monitor user outcomes over time. Such studies would offer valuable insights into the lasting benefits of DFRS, including its potential influence on other health aspects, like cardiovascular health and overall quality of life. By exploring these areas, we can better assess the long-term viability of DFRS as a supportive tool for managing diabetes.

However, DFRS improves the precision and efficiency and the dietary advice through the data formulary approach; thus, it improves the health care delivery. DFRS has shown initial effectiveness; however, its integration must be experimentally verified with different patient populations and providers’ environments. More extended research is required to evaluate the long-term consequences of DFRS on different aspects of diabetes care and functionality and understand how it may be incorporated with other aspects of diabetes care such as medication and physical activity regimes. However, continuous improvement and development of DFRS are crucial to fully realizing its capabilities and evolving usable, innovative approaches for chronic disease diagnosis and treatment by applying personalized medicine. This study is a powerful resource that enables clients to develop effective and helpful dietary strategies in Diabetes Mellitus and characteristic patient satisfaction and holistic healthcare delivery. Elaborate combining modern machine learning techniques and a user-centered approach, DFRS enables the person with diabetes to make proper food choices and keep healthy. The future research should be aimed at increasing effective patients’ participation and compliance with the prescribed diet. This is as far as designing interfaces that are easy to use, adopting gamification strategies in order to encourage users, and feedback mechanisms to improve interaction. To gain an understanding of such strategies is critical to enhance the usability of dietary management frameworks such as the DFRS. Furthermore, subsequent research should examine how the DFRS may be incorporated with medication regimens and other aspects of diabetes self-management. This could be in the form of compiling intensive packages of Guidelines that include but are not limited to Calorie Plans and Medication Schedules coupled with Real-time glucose levels and Patients’ Lifestyles. Studying the interaction between diet control and drug treatment will be essential in providing comprehensive client care for patients with diabetes. The study acknowledged its limitation in the number of patby ients recruited limiting age, gender, and ethnicity diversity in their sample population and intends to capture a more representative patient population in future studies. This will allow the DFRS to consider and respond to the recommendations for a broader population group, and test the applicability of the system for different subtypes of diabetes and per different demographical characteristics. Also, recommendations in a culturally competent manner for patient involvement and compliance will be added.

## Supporting information

S1 File The information stripped of the patients’ identifiers – thirty demographic, medical, and clinical characteristics relating to 1,000 patients with diabetes. This set of data is indispensable when it comes to creating a customized diet to patients who have diabetes.
(CSV)

S2 File A large scale food dataset containing the nutrient profiles of 2,540 foods, compiled from the different authoritative sources including USDA FoodData Central. This dataset is important the provide correct and diversified diet recommendations to many users.
(CSV)

## Acknowledgments

The author acknowledges the invaluable contributions of all individuals and organizations involved in the data collection process for this study, particularly the USDA FoodData Central for providing access to comprehensive nutritional information.
